# Evaluation of the Effect of *Cichorium intybus* L. on the Liver Enzymes in Burn Patients: A Randomized Double-Blind Clinical Trial

**DOI:** 10.1155/2024/1016247

**Published:** 2024-01-11

**Authors:** Abdolkhalegh Keshavarzi, Rahimeh Akrami, Mohammad M. Zarshenas, Saeid Zareie, Tayyeb Ghadimi, Ali Najafi, Mahsa Rostami Chijan, Azizallah Dehghan, Elham Zarenezhad

**Affiliations:** ^1^Shiraz Burn and Wound Healing Research Center, Shiraz University of Medical Sciences, Shiraz, Iran; ^2^Traditional Medicine and Hydrotherapy Research Center, Ardabil University of Medical Sciences, Ardabil, Iran; ^3^Department of Phytopharmaceuticals (Traditional Pharmacy), School of Pharmacy, Shiraz University of Medical Sciences, Shiraz, Iran; ^4^Nurse of Intensive Care Unit (ICU) of Amir Al-Momenin Burn Injury Hospital, Shiraz University of Medical Sciences, Shiraz, Iran; ^5^Department of Plastic and Reconstructive Surgery, School of Medicine, Iran University of Medical Sciences, Tehran, Iran; ^6^Department of Persian Medicine, Fasa University of Medical Sciences, Fasa, Iran; ^7^Noncommunicable Diseases Research Center, Fasa University of Medical Sciences, Fasa, Iran

## Abstract

Burn injuries are considered an important public health problem in the world. Burns are considered the fourth most common kind of trauma in the world, after traffic accidents, falls, and interpersonal violence. Various biochemical agents are involved in the burn healing process such as cytokines (such as IL-6 and TNF-*α*), antioxidants, and liver and kidney damage biomarkers. *Cichorium intybus* L. and milk thistle extracts showed a wide range of pharmacological activities such as significant antimicrobial effect and antioxidant activity, as well as anti-inflammatory, antidiabetic, antiproliferative, antiprotozoal, and hepatoprotective effect. Also, these two herbs possess blood-cleansing, detoxifying, laxative, and invigorating activities. Some research confirmed that the preparations of the extract are very suitable for the treatment of nonalcoholic fatty liver disease. This is a double-blind randomized controlled clinical trial. Patients with 2nd and 3rd degree burns have been selected to participate in the study according to the inclusion criteria. A total of 60 patients were selected and divided into intervention and control groups (30 patients in each group). Patients in the intervention group received chicory seed syrup 10 cc three times a day and 1 placebo capsule, and those in the control group received placebo syrup (10 cc three times a day) and one Livergol (140 mg of silymarin in each capsule) capsule. Lab data such as liver function tests, albumin, creatinine, BUN, and hemoglobin were checked every 3 days and 1 week after discharge. The treatment lasted for 4 weeks. According to the results of the study, although the average of liver enzymes at the end of the study does not show a significant difference between the two groups, the level of liver enzymes in each group decreased on the 15th day of the study compared to the first day. This trial is registered with IRCT20180609040016N1.

## 1. Introduction

Burn injuries are considered an important public health problem in the world. Burns are considered the fourth most common kind of trauma in the world, after traffic accidents, falls, and interpersonal violence [1–3]. Burn injuries can be caused by cold, chemical compound, heat, electric source, and radiation. But most of them are caused by fire or hot liquids [4]. Almost 90% of burns occur in developing countries. Some research reported that some parameters such as the economic, social, and cultural level of the society influence the type and quantity of burn [5]. According to a WHO report, at least 3000000 people die from burns annually [6]. In Iran, burn injury is ranked thirteen in the burden of diseases with the highest rank of morbidity and mortality [7].

Different risk factors including male gender [8], crowding [9], and poverty and age (under 4 and over 65 years) have been reported [10]. Various serum enzymes such as AST, ALT, GGT, and ALP contribute to the function of liver cells; when the cells undergo necrosis, the concentration of these enzymes increases [11]. Also, serum CHF is formed by the liver; decrease in this enzyme demonstrates liver damage [12]. Various biochemical agents are involved in the burn healing process such as cytokines (such as IL-6 and TNF-*α*), antioxidants, and liver and kidney damage biomarkers [13].

Natural compounds have been used for hundreds of years to control burn injury. Medicinal plants with a wide range of biological effects have received more attention [14–16]. Common chicory (*Cichorium intybus* L.) is a somewhat woody plant belonging to the Asteraceae family usually with blue flowers [17]. A methanol extract of *C. intybus* showed a wide range of pharmacological activities such as significant antimicrobial effect and antioxidant activity [18], as well as anti-inflammatory, antidiabetic, antiproliferative, antiprotozoal, and hepatoprotective effect. Also, the herb possesses blood-cleansing, detoxifying, laxative, and invigorating activities [19].

Similarly, as a popular plant from the family Asteraceae, *Silybum marianum* L. (milk thistle), with the main bioactive compound as silymarin [20], is a medicinal plant which has been used in various liver diseases for centuries [21]. Some research confirmed that the extracts or preparations of milk thistle extract are very suitable for the treatment of nonalcoholic fatty liver disease [21–23]. According to sources of Persian medicine, both mentioned plants are used to treat various liver diseases [24]. However, due to the low bioavailability of *C. intybus*, investigation of the potential hepatoprotective effects of other generation of this family could be of interest. Accordingly, this research aimed to evaluate the effect of *C. intybus* on liver enzyme variation in burn patients.

## 2. Materials and Methods

### 2.1. Study Design and Participants

This is a double-blind randomized controlled clinical trial. Participants were selected among hospitalized patients referred to Amir al-Momenin Hospital. The inclusion criteria included age between 20 and 75 years, having 2nd and 3rd degree burns, extent of burns between 10 and 50%. The burn time for patients is less than two days. Their burns are between 10% and 50% body surface percentage. Exclusion criteria are as follows: the patients are not intubated, no history for liver disease, hyperlipidemia, and seizure, and no pregnancy.

After interviewing and informing the patient, all patients enter the study after filling the informed consent form. Initially, personal and demographic information forms are filled out for all participants. 10 cc of blood was taken from all patients. Initial tests, including aspartate aminotransferase (AST), alanine transaminase (ALT), alkaline phosphatase (ALP), albumin, creatinine (Cr), blood urea nitrogen (BUN), and blood sugar (Bs), were checked by using the AutoAnalyzer device (Mindray-Bs-380 model). White blood cell count (WBC), hemoglobin (Hb), and platelet (Plt) were checked by using the cell counter device (Nihon Kohden Celltac ∝ MEK-6510k model). Also, the i-Smart 30 PRO Electrolyte Analyser is used for the quantitative measurements of sodium (Na) and potassium (K).

Then the patients were divided into intervention and control groups. Patients in the intervention group received chicory seed syrup and placebo capsules, and those in the control group received placebo syrup and Livergol capsules (which are standardized based on the presence of 140 mg of silymarin in each capsule purchased from Gol Daru factory, Isfahan, Iran). Patients in both groups received medicine and placebo until discharge, and liver enzymes were checked every three days and one week after discharge.

**Figure 1:**
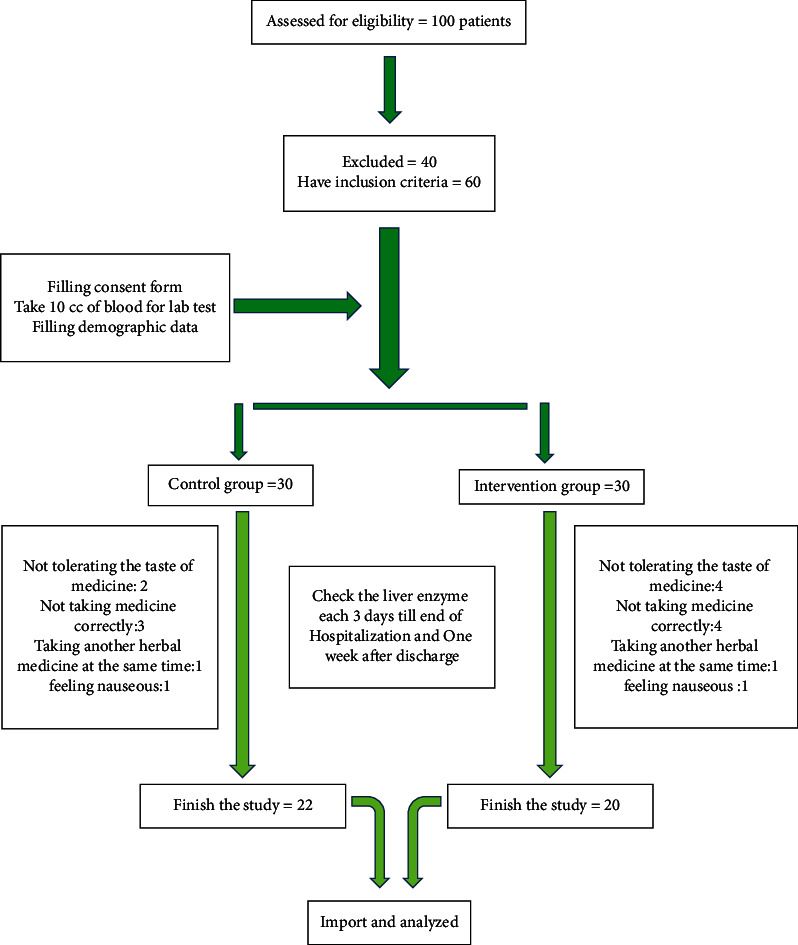
CONSORT flowchart.

During the study, both groups of patients received standard burn treatments and were visited by a doctor every day, and if side effects occurred, the treatment is stopped and the patient was withdrawn from the plan. The CONSORT flowchart is illustrated in Figure 1.

This study was approved by the ethics committee of Shiraz University of Medical Sciences (Code: IR.SUMS.REC.1396.194) and has been registered in Iranian Registry of Clinical Trials (irct.ir) with the code number IRCT20180609040016N1.

### 2.2. Randomization and Blinding Method

The enrolled patients were divided into two groups: A (intervention) and B (control). To select the group, the balanced block randomization method with blocks of four has been used. Before the start of the study, it was determined which intervention group and which control group would be placed by tossing a coin. The patient and the researcher did not know about the nature of the groups.

Totally, 100 g of chicory seed powder was boiled in one liter of distilled water for 6 to 10 minutes. Subsequently, the yielded extract was filtered and concentrated with 50% sucrose (sugar) syrup and packed in 250 cc containers (represented as syrup A). The dose for the patients was adjusted to 10 cc three times a day, based on the traditional reported dose [25]. Based on the calculations made, when the syrup is prepared in this way, according to the amount of syrup consumed during the day, each person has consumed approximately 6 g of chicory seeds during the day.

Placebo syrups (syrup B) contained a sugar syrup with 1% of the extract. All syrup bottles were completely similar in terms of color, shape, and size.

On the other hand, placebo capsules (capsule A) contained 140 g of roasted corn flour. The control group received capsules containing dry *Silybum marianum* extract (capsule B), which are standardized based on the presence of 140 mg of silymarin in each capsule (purchased from Gol Daru factory, Isfahan, Iran). All capsules were completely similar in terms of color and appearance. The daily dose was one capsule two times a day.

The control group received capsule B and syrup B, and the intervention group received capsule A and syrup A.

### 2.3. Sample Size

According to the study error of 0.05 and the power of 80% and considering the pilot study the mean of AST before and after the intervention in control and intervention groups which were obtained 124.22 ± 165.133 and 38 ± 31.56, respectively, the suitable sample size for each group is 29 person was calculated, and independent sample T test and Generalized Estimating Equations (GEE) were used for analysis.

## 3. Result

### 3.1. General and Demographic Data

A total of 60 patients were enrolled in the study.

Finally, 42 patients (20 women and 22 men) with mean age of 40.5 years completed the study.

The comparison of the baseline demographic criteria, hemoglobin, WBC, platelet, BUN, Cr, Na, K, and BS between the 2 groups shows no statistically significant difference (Table 1).

As shown in Table 2, at the beginning of the study, albumin, ALT, AST, and ALP showed no difference in both groups (Table 2).

According to the results of the study, although the average of liver enzymes at the end of the study does not show a significant difference between the two groups (Table 3), according to the graphs, the level of liver enzymes in each group decreased on the 15th day of the study compared to the first day, but it was not significant (Figure 1).

### 3.2. Side Effects

No specific side effects were observed in the two groups. One person in each group reported feeling nauseated and 2 patients in the control group and 4 patients in the intervention group did not tolerate the taste of medicine.

## 4. Discussion

Injuries caused by burns, especially in the age group of children, are very stressful and can even lead to death [26]. Maintaining hemostasis and liver metabolism is very important in patients with severe burns. There are many evidences that the liver undergoes hypertrophy after severe burns. However, there is insufficient evidence for the extent and duration of liver enlargement [1].

There are varying degrees of liver damage after thermal damage, which depends on the severity of thermal damage. Thermal injuries can cause liver damage by different mechanisms, such as reducing perfusion and producing proinflammatory cytokines, or activating cell death signals, edema formation, and lipid changes [26].

Many studies have been conducted on the effect of *Silybum marianum* plant, which show the protective effects of this plant on the liver disorder such as acute viral hepatitis, toxin and drug-induced hepatitis, alcoholic liver disease, and chronic hepatitis/cirrhosis [3]. Based on some studies, use of *Silybum marianum* in patients with acute viral hepatitis for at least 3 weeks can reduce increase of liver enzymes [27]. Flora et al.'sstudy shows that the use of *Silybum marianum* in patients with acute alcoholic hepatitis makes the process of enzyme normalization happen faster [27]. There are many studies that show that this plant can be effective for fatty liver, cirrhosis, and hepatitis following drugs and toxins [26, 28–32]. Some studies showed that silymarin is the active component of *Silybum marianum* and it can inhibit the growth of hepatocellular carcinoma HepG2 cell line, inducing apoptosis to increase the cell population in the phase below G0/G1. Mechanistically, treatment with silymarin reduced mitochondrial transmembrane potential, leading to an increase in cytosolic cytochrome c (Cyt c), downregulating proliferation-associated proteins (PCNA, c-Myc, cyclin D1, and *β*-catenin) and antiapoptotic proteins (survivin and Bcl-2), and upregulating proapoptotic proteins (caspase-3, Bax, APAF-1, and p53). The hepatoprotective effect on NAFLD is improving by increasing the levels of ALT, AST, TC, TG, HDL-C, and LDL-C, decreasing protein expressions of IL-6, MAPK1, Caspase 3, p53, and VEGFA, and increasing protein expression of AKT1. [33–35].

Two trials have shown that silymarin can prevent elevated liver enzymes after cholecystectomy in patients anesthetized with halothane [36] or with fentanyl [37].

Based on Ahmed et al.'s study [38] and other similar studies that show the positive effects of milk thistle in controlling liver enzymes, the use of capsules containing dry *Silybum marianum* extract is considered as a treatment in the control group. Therefore, we were able to compare the effect of chicory seeds in controlling liver enzymes with *Silybum marianum*.

Chicory showed antioxidant, antibacterial, antipyretic, antidiabetic, antihepatotoxic, anti-inflammatory, antiulcerogenic (root), antihyperglycemic, antihyperlipidemic, anticancer, and antimalarial activities. Also, inulin from the plant can be used as a probiotic that protects the intestinal flora and can reduce fasting serum glucose (FSG), HbA1C, AST, and ALP, serum calcium significantly [39, 40].

Based on the study of Faraji et al., the use of brewed chicory leaf made from 15 g chicory leaf after 6 weeks can have positive effects in reducing liver enzymes (AST and ALT) and total bilirubin in patients with nonalcoholic fatty liver disease [40]. In the study of Marzban et al., it was found that the consumption of aqueous extract of chicory seeds for 12 weeks can significantly reduce liver enzymes in patients with nonalcoholic fatty liver disease [41]. As seen in our study, the effect of chicory seed syrup has prevented the rise of liver enzymes in burn patients for 18 days, which can point to the protective role of this syrup on the liver.

In many animal studies where the protective effect of chicory seed has been investigated, the reduction of liver enzymes is clearly visible [38, 42, 43].

Based on the results of our study, as expected, liver enzymes decreased during treatment in both groups, which is similar to studies that have been conducted.

## 5. Conclusion

Our findings show that both silymarin and chicory seed treatments have been effective in reducing liver enzymes in burn patients. In conclusion, these treatments can be useful when the body is under the influence of strong stress such as burns.

## 6. Limitation

The problems of the study included the small sample size and dropout of patients. To achieve better results, designing studies with a higher sample size is needed. Also, prolonging the duration of the study can be effective to prove the effects of these two medicinal plants.

## Figures and Tables

**Table 1:** The baseline and demographic variables in intervention and control groups.

Variables	Group	Number	Percent	*P* value	
Gender	Control	Male: 13	59.1		0.537
		Female: 9	40.9		
	Intervention	Male: 9	45		
		Female: 11	55		

	Group	Number	Mean	SD	*P* value

Age (year)	Control	22	40.1364	10.02993	0.849
	Intervention	20	40.8750	13.66687	0.856

Hemoglobin (g/dl)	Control	22	15.0714	0.49829	0.572
	Intervention	20	14.6800	0.47064	0.571

WBC^1^ (4.5 to 11.0 × 10^9^/L)	Control	22	14.0678	1.17767	0.287
	Intervention	20	12.4950	0.83466	0.283

Platelet (150 to 450 × 10^9^/L)	Control	22	278.3333	17.56530	0.236
	Intervention	20	313.6000	23.72700	0.240

BUN^2^ (mg/dL)	Control	22	13.7143	1.07288	0.277
	Intervention	20	11.9900	1.13883	0.277

Cr^3^ (mg/dL)	Control	22	1.0048	0.04363	0.997
	Intervention	20	1.0050	0.03657	0.997

Na^4^ (mEq/L)	Control	22	139.7143	0.69742	0.236
	Intervention	20	138.5000	0.73090	0.237

K^5^ (mEq/L)	Control	22	3.7667	0.06223	0.327
	Intervention	20	3.8850	0.10318	0.334

Bs^6^ (mg/dL)	Control	22	121.7619	4.50339	0.291
	Intervention	20	137.5500	14.36140	0.305

^1^White blood cell. ^2^Blood urea nitrogen. ^3^Creatinine. ^4^Blood sodium. ^5^Potassium. ^6^Blood sugar.

**Table 2:** The baseline lab data variables in two groups.

Variables	Control (mean)	Intervention (mean)	*P* value
Albumin (g/L)	3.8318	3.6450	0.320
ALT^1^ (U/L)	42.3636	31.8421	0.541
AST^2^ (U/L)	50.3636	35.2105	0.421
ALP^3^ (U/L)	208.1364	237.1053	0.229

^1^Alanine transaminase. ^2^Aspartate aminotransferase. ^3^Alkaline phosphatase.

**Table 3:** Mean of variables between two groups.

Variables	Times of testing	Mean	*P* value
Albumin (g/L)			
Control	132	3.2	0.7
Intervention	90	2.9	
ALT^1^ (U/L)			
Control	80	49.2	0.220
Intervention	66	41.04	
AST^2^ (U/L)			
Control	80	42.8	0.241
Intervention	66	37.8	
ALP^3^ (U/L)			
Control	78	278.26	0.388
Intervention	55	292.41	

^1^Alanine transaminase. ^2^Aspartate aminotransferase. ^3^Alkaline phosphatase.

## Data Availability

The datasets used and analyzed during the current study are available from the corresponding and first authors upon reasonable request.

## Supplementary Materials

Supplementary Materials Supplementary Figure 1: mean of aspartate aminotransferase (AST) at the first and 15th day between two groups. Supplementary Figure 2: mean of alanine transaminase (ALT) at the first and 15th day between two groups.
