# Supplementary material for: Evaluation of the Effect of Cichorium intybus L. on the Liver Enzymes in Burn Patients: A Randomized Double-Blind Clinical Trial
Source: Int J Clin Pract. 2024 Jan 11;2024:1016247. doi: 10.1155/2024/1016247 (PMC10796187; doi:10.1155/2024/1016247)
Supplement: Supplementary Materials — Supplementary Figure 1: mean of aspartate aminotransferase (AST) at the first and 15th day between two groups. Supplementary Figure 2: mean of alanine transaminase (ALT) at the first and 15th day between two groups. [file 1016247.f1.zip › Figure 2 (1).docx]

**Figure 2: mean of Alanine Transaminase (ALT) at the first and 15th day between two groups**
